# Relative contribution of climate and non-climate drivers in determining dynamic rates of boreal birds at the edge of their range

**DOI:** 10.1371/journal.pone.0224308

**Published:** 2019-10-24

**Authors:** Michale J. Glennon, Stephen F. Langdon, Madeleine A. Rubenstein, Molly S. Cross

**Affiliations:** 1 Wildlife Conservation Society, Saranac Lake, NY, United States of America; 2 Shingle Shanty Preserve and Research Station, Long Lake, NY, United States of America; 3 National Climate Adaptation Science Center, U.S. Geological Survey, Reston, VA, United States of America; 4 Wildlife Conservation Society, Bozeman, MT, United States of America; University of Waikato, NEW ZEALAND

## Abstract

The Adirondack Park in New York State contains a unique and limited distribution of boreal ecosystem types, providing habitat for a number of birds at the southern edge of their range. Species are projected to shift poleward in a warming climate, and the limited boreal forest of the Adirondacks is expected to undergo significant change in response to rising temperatures and changing precipitation patterns. Here we expand upon a previous analysis to examine changes in occupancy patterns for eight species of boreal birds over a decade (2007–2016), and we assess the relative contribution of climate and non-climate drivers in determining colonization and extinction rates. Our analysis identifies patterns of declining occupancy for six of eight species, including some declines which appear to have become more pronounced since a prior analysis. Although non-climate drivers such as wetland area, connectivity, and human footprint continue to influence colonization and extinction rates, we find that for most species, occupancy patterns are best described by climate drivers. We modeled both average and annual temperature and precipitation characteristics and find stronger support for species’ responses to average climate conditions, rather than interannual climate variability. In general, boreal birds appear most likely to colonize sites that have lower levels of precipitation and a high degree of connectivity, and they tend to persist in sites that are warmer in the breeding season and have low and less variable precipitation in the winter. It is likely that these responses reflect interactions between broader habitat conditions and temperature and precipitation variables. Indirect climate influences as mediated through altered species interactions may also be important in this context. Given climate change predictions for both temperature and precipitation, it is likely that habitat structural changes over the long term may alter these relationships in the future.

## Introduction

The Adirondack Park in New York State is in the southern edge of the range for several species of boreal forest birds within eastern North America. The network of public and private lands that constitutes The Adirondack Park represents critical boreal habitat for both resident and migratory birds, including large patches of lowland boreal habitats which are otherwise rare in the northeastern United States. The habitats of these boreal specialists—cool, wet, sphagnum-draped bogs and forested northern swamps—are thought to be particularly vulnerable to climate change [1,2]. Effects may include encroachment by trees into open bog landscapes [3,4], increased competition with southern plant and animal species expanding northward [5], and altered timing of annual events like insect emergence [6]. In addition to climate-driven changes to the boreal habitat, these boreal specialists may experience direct effects of climate change, including the potential for reduced winter mortality under more benign winter conditions [7] or increased mortality during extreme weather events (e.g., cold snaps [8]). As northern species, adapted to northern habitats and climates living at the southern edge of their range, they are expected to be vulnerable to a warming climate, and to serve as useful indicators of potential changes to come across the species’ full distributions.

Non-climate stressors, including changing land use patterns, altered wetland size and connectivity, and exurban residential development, are also significant drivers of change in this region. Distinguishing climate-driven changes from other drivers is particularly challenging in this context, but is a critical component of identifying the underlying reasons for declines in a number of boreal species. Species interactions are believed to be more influential at southern ends of species ranges, whereas climate is expected to play a greater role at northern edges [9,10]. As such, we may also expect that the influence of climate change on Adirondack boreal birds at the southern edge of their range may be via indirect mechanisms such as increased competition and predation.

This analysis uses a decade of occupancy data (2007–2017) to identify the primary drivers of dynamic occupancy rates (colonization and extinction) for eight species associated with lowland boreal habitats in the Adirondack Park. We explore the relative contributions of climate and landscape characteristics on boreal bird occupancy patterns. A previous analysis revealed declining occupancy patterns for 4 of these species, and only one of them demonstrating a pattern of increase [11]. We previously explored the influence of landscape-level drivers on boreal bird dynamics, investigating the effects of wetland size, connectivity, latitude, elevation, and human footprint on their occupancy patterns in low boreal peatlands in the Adirondacks [11]. Overall, boreal birds were more likely to disappear from smaller, isolated wetlands located in close proximity to roads and residential development [11]. With respect to possible climate-driven patterns, the influence of latitude and elevation on the dynamics of these species was variable. Some species appeared to demonstrate northward or upslope movements–climate-driven responses documented for numerous species around the globe [12]–but latitude and elevation may provide poor proxies for occupancy patterns driven by climate change in this system. The largest, most well-connected boreal wetland complexes in the Adirondacks are located at high latitude and low elevation in the northwest region of the park. Within our study sites, elevation and latitude are negatively associated and, as such, northward latitudinal movements may be associated with changes in elevation as well as changes in temperature and precipitation.

The present analysis builds upon the previous 5-year analysis [11] by using data from 2012–2016 to examine longer-term (decadal) trends in occupancy, and to more fully address the degree to which climate change may drive observed changes in boreal bird occupancy. We explore the influence of temperature and precipitation on the long-term spatial occupancy patterns of 8 boreal bird species in the Adirondacks. We reexamine the previously analyzed non-climate drivers with a longer-term, 10-year dataset and we specifically compare how temperature and precipitation influence dynamic rates in comparison to the landscape context variables previously investigated. Our questions were: (1) were previously identified occupancy patterns maintained over the longer term, (2) how do temperature and precipitation, specifically, influence occupancy dynamics of boreal birds in Adirondack peatlands, and (3) how do temperature and precipitation influence dynamic occupancy patterns in comparison to known influences of non-climate drivers including wetland size, connectivity, and human footprint? We expected that boreal birds would conform to expectations of metapopulation biology given the patchy nature of their habitats in the Adirondacks, and therefore, size and connectedness of their wetland habitats would positively influence colonization and persistence rates. We also expected that, given their location at the southern range extent within eastern North America and in a geographic zone that lies at the transition between the temperate and the boreal, climate would be an important potential influence on their long-term dynamics. This effort builds upon the previous investigation of these species by examining climate directly through the influence of temperature and precipitation as potential drivers of dynamic occupancy patterns.

## Methods

### Target species

Our non-invasive sample method required no specific research permits. Nearly all sites were located on public land; permission for access was obtained on the small number of private land sites. Our study site, target species, site selection, and monitoring methods are fully described in [11]. Our analysis focuses on 8 focal species at or close to the southern extent of their eastern North American range in the Adirondack Park and all known to occur in the Canadian boreal including black-backed woodpecker (BBWO; *Picoides dorsalis*), boreal chickadee (BOCH; *Poecile hudsonicus*), Canada jay (CAJA; *Perisoreus canadensis*), Lincoln’s sparrow (LISP; *Melospiza lincolnii*), palm warbler (PAWA; *Setophaga palmarum*), olive-sided flycatcher (OSFL; *Contopus cooperi*), rusty blackbird (RUBL; *Euphagus carolinus*), and yellow-bellied flycatcher (YBFL; *Empidonax flaviventris*). These species were selected because they best represent the lowland boreal habitats of the Adirondacks and are also well-sampled with point-count methods.

### Study site locations

**Fig 1:**
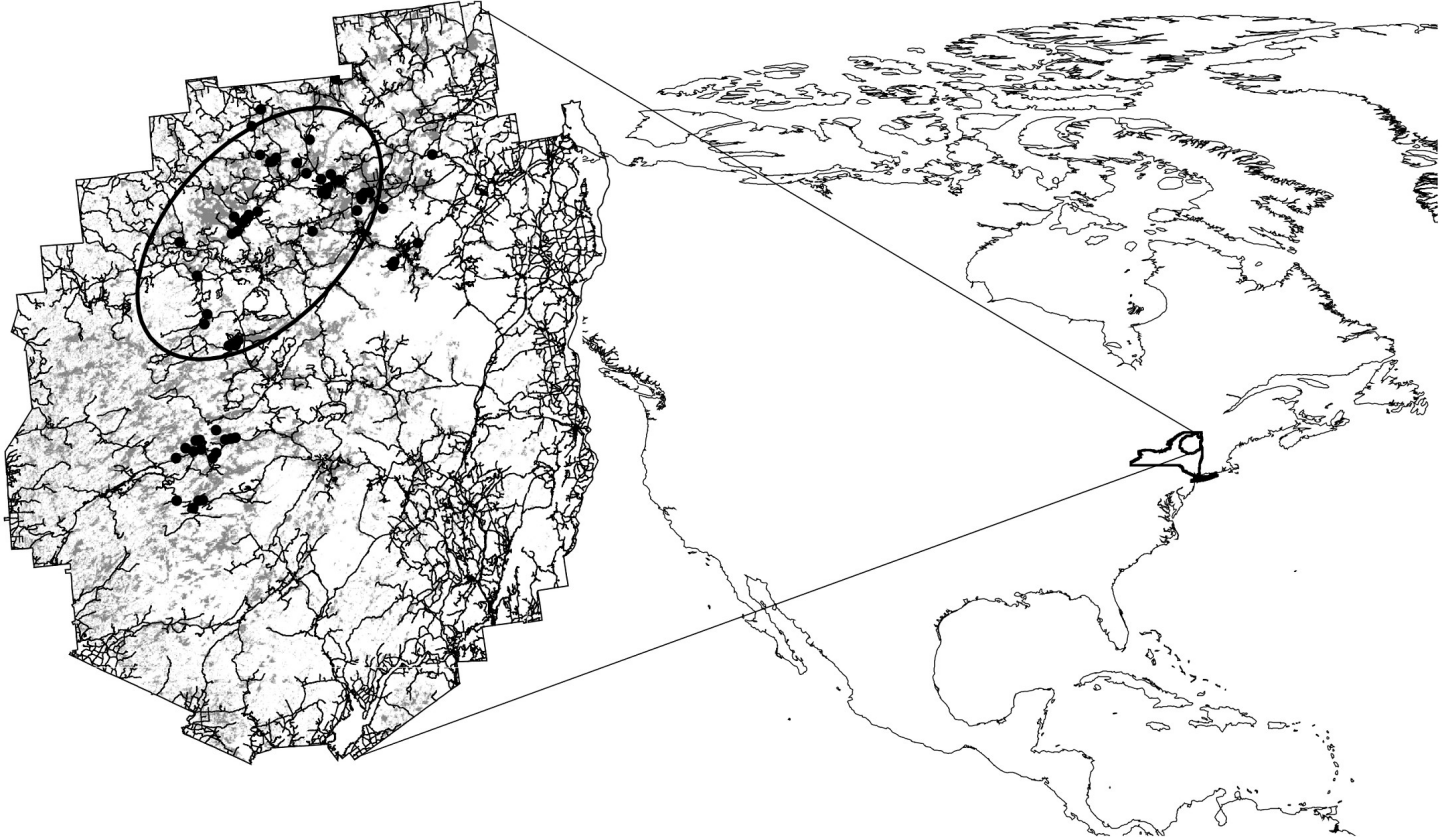
Location of the Adirondack Park in northern New York State and North America, depicting study locations (black dots), low elevation boreal habitat (grey), the road network, and the region of the park (oval) where the largest open bog complexes are located, often referred to as the “boreal core.”.

Our study was conducted in the Adirondack Park, an area of 19,700 km^2^ located in the northern part of New York State in the US (43˚58’14” N, 74˚03’12” W). The boreal habitats that are the subject of this study consist of bogs, fens, wooded wetlands, and open river corridors in the Adirondack Park. Boreal habitats are distributed in small patches throughout the Adirondacks but are most concentrated in a band running from the north-central part of the park to the southwestern edge (Fig 1). These habitats contain both high- and low-elevation components; our work deals solely with low-elevation boreal communities (mean 507 m, range 122–1250 m) and does not address the montane boreal (mean 898 m, range 556–1583 m), which is the focus of a separate high-elevation bird monitoring program [13]. As recently characterized for the Northeast region [14], boreal communities in the Adirondacks fall primarily into Northern Swamp, Northern Peatland, and Boreal Upland Forest macrogroups, with dominant habitat types within those macrogroups including Northern Appalachian Acadian Conifer Hardwood Acid Swamp, Boreal Laurentian-Acadian Acidic Basin Fen, Boreal Laurentian Bog, and Acadian Low Elevation Spruce Fir Forests and Sub-Boreal Spruce Flats. These are wet, acid, carbon-accumulating habitats that range from forested to open sites, with mean summer temperature < 18° C and predominantly coniferous vegetation. Dominant vegetation includes tree species such as black spruce (*Picea mariana*) and tamarack (*Larix laricina*), ericaceous shrubs such as leatherleaf (*Chamaedaphne calyculata*) and Labrador tea (*Ledum groenlandicum*), herbaceous plants such as pitcher plant (*Sarracenia purpurea*) and sedges (e.g., *Carex* spp., *Eriophorum* spp.) and *Sphagnum* mosses.

### Sampling

We conducted unlimited distance point counts to assess presence/absence of our 8 target species along transects of 5 points spaced at least 250m apart within boreal wetland habitats [15]. We employed spatial replication of sample points rather than temporal replication, to reduce costs and allow for the calculation of detection probabilities [16,11]. The sites themselves, and not the five points within each site, serve as the experimental units for the purposes of analysis. All points were surveyed for 10-minutes between the hours of 5:00 and 9:00 am during the primary breeding season on survey dates ranging from the last week of May to the third week of July, with the majority of sites sampled in June. Surveys were conducted by trained observers, the majority of whom conducted counts for 3 or more of the project years. During counts, we recorded the date, start and end time for each survey, ambient temperature, and sky and wind conditions. We have sampled more than 80 locations over the course of the project; 58 of those were sampled consistently for the period between 2007 and 2016 and are the subject of the prior [11] and current analysis.

### GIS datasets

GIS datasets used previously for investigating non-climate drivers of boreal bird dynamics were employed in the current study to examine the influence of wetland size (area), connectivity, human footprint, latitude, and elevation on colonization and extinction rates. These datasets have been previously described [11] and are described only briefly here. Area and connectivity of study wetlands were calculated from wetland cover type data in the form of polygon vector files provided by the Adirondack Park Agency (APA) which classify all park wetlands by system and class [17]. The cluster and outlier analysis tool in ArcMap 10 was used to calculate a Moran’s *I* value for boreal wetland polygons with a fixed distance band of 5 km; these values were then used as an index of habitat connectivity for boreal wetland habitats. Area and connectivity of study wetlands were calculated previously as described in [11]. Similarly, we again used latitude and elevation information for each study location from the previous analysis, as well as human footprint [18] scores averaged across each of the 5 points along each study transect to characterize human impact at each study wetland [11]. Although the regional human footprint map [18] was created a decade ago, we used it to be consistent with the previous analysis and because we believe that the broad-scale features mapped within it (e.g., population density, urban areas, roads, rail lines, energy infrastructure) have not changed appreciably within the Adirondack region since the time of its publication.

### Temperature and precipitation

Temperature and precipitation data were obtained from the Parameter-elevation Relationships on Independent Slopes model (PRISM, [19]) at 800m resolution. We obtained mean temperature and precipitation values for study site locations for all months and years between December 2006 and August 2016 and used them to calculate mean winter (December–March) and breeding (May–August) season temperature, variability in winter and breeding season temperature, mean winter and breeding season precipitation, and variability in winter and breeding season precipitation. We also calculated the annual mean temperature of the coldest and warmest month, and mean precipitation of the wettest and driest month during the study period (2007–2016).

### Non-climate drivers

As per the prior 5-year analysis, to investigate dynamics of boreal birds in the Adirondacks, we used the multi-season model implemented in program Presence [20] to calculate detection (p), occupancy (ψ), colonization (γ), and extinction (ε) probabilities for 2007–2016 for each target species.

We repeated a model set tested previously [11] to examine the influence of wetland size, connectivity, latitude, elevation, and human footprint on boreal bird occupancy. We first modeled detection for each species while holding other parameters constant and tested 6 variables for their influence on detection probability including wind, sky (relative cloud cover), date, time, temperature, and observer. We used the default parameterization of the multi-season model, which estimates initial occupancy, colonization, and extinction probabilities directly, and is the most numerically stable [16]. Occupancy rates for years 2–10 were calculated from initial ψ, γ, and ε. When analyzing the influence of non-climate drivers on colonization/extinction rates, we held one rate constant and varied the other within the model set, resulting in a set of 11 models for each species (Table 1). Because non-climate drivers (i.e., wetland size, elevation, human footprint etc.) do not vary significantly over the timescale of our study, we modeled colonization and extinction rates as constant over time but varying spatially with characteristics of individual study sites. We drew inferences from the betas and model-averaged estimates of γ and ε for all models and used factor weights to explore the relative importance of each covariate across all species [21,22]. We expected that wetland area and connectivity would remain positive influences on colonization and persistence and that human infrastructure would remain a negative influence on colonization and persistence–expecting that the majority of these specialist birds would continue to persist in larger, more connected wetlands and favor those farther from human disturbance and habitat alteration. Given the prior mixed signals for the influence of latitude and elevation [11], we made no predictions about these drivers and instead directly explored potential climate change drivers in the second part of our analysis by including temperature and precipitation specifically. We used model-averaged estimates of colonization and extinction to calculate occupancy rates for each of the years between 2007 and 2016 in order to examine trends over time and the degree to which trends and drivers were consistent with the 5-year analysis when extended to the 10-year dataset.

**Table 1:** Eleven models used to predict probability of occupancy (ψ), colonization (γ), and extinction (ε) for 8 bird species in boreal wetlands of the Adirondack Park, NY, 2007–2016.

Model	Predicted dynamics dependent on
ψ (.), γ(.), ε(.)	Constant rates of colonization and extinction.
ψ (.), γ(Wetland Area), ε(.)	Area-driven colonization rates.
ψ (.), γ(Connectivity), ε(.)	Connectivity-driven colonization rates.
ψ (.), γ(Latitude), ε(.)	Latitude-driven colonization rates.
ψ (.), γ(Elevation), ε(.)	Elevation-driven colonization rates.
ψ (.), γ(Human Footprint), ε(.)	Human impact-driven colonization rates.
ψ (.), γ(.), ε(Wetland Area)	Area-driven extinction rates.
ψ (.), γ(.), ε(Connectivity)	Connectivity-driven extinction rates.
ψ (.), γ(.), ε(Latitude)	Latitude-driven extinction rates.
ψ (.), γ(.), ε(Elevation)	Elevation-driven extinction rates.
ψ (.), γ(.), ε(Human Footprint)	Human impact-driven extinction rates.

### Climate drivers

We used a second model set to explore the influence of temperature and precipitation on dynamic rates. We used PRISM data (800m) to explore the influence of means, variability, and extremes of breeding and wintering season temperature and precipitation on colonization and extinction rates for 8 boreal bird species. Specific variables included: mean winter (December–March; Winter Temp) and breeding season (May–August; Breeding Temp) temperature, variability in winter (Var Winter Temp) and breeding season (Var Breeding Temp) temperature, mean winter (Winter Ppt) and breeding season (Breeding Ppt) precipitation, variability in winter (Var Winter Ppt) and breeding season (Var Breeding Ppt) precipitation, mean temperature of the hottest (Temp Hottest Month) and coldest (Temp Coldest Month) months, and mean precipitation during the wettest (Ppt Wettest Month) and driest (Ppt Driest Month) months. As per the initial analysis, we held one of the dynamic rates constant and varied the other within the model set. We found that means and extremes were correlated in terms of temperature during the time period of our study, but not in terms of precipitation. Given that each of our models contained only one predictor variable, we chose to maintain both aspects of temperature information in the model set. In contrast to the landscape context variables, climate drivers are expected to display comparatively high interannual variability. We therefore modeled each of 12 climate variables in two ways; one in which these variables changed annually and were related to dynamic rates for each individual year, and one in which conditions were averaged across the 10-year study period and dynamic rates were related to long-term average conditions. This combination of 12 climate variables modeled on each of 2 dynamic rates and as both seasonal and average effects resulted in a total of 48 total models conducted for each species (Table 2). Though 5 of our target species are migratory, winter conditions can affect timing of food resources [23] and conditions of the vegetation for nesting in the subsequent breeding season [24] and, as such, we modeled all climate variables for all species.

**Table 2:** Models used to predict probability of occupancy (ψ), colonization (γ), and extinction (ε) for 8 bird species in boreal wetlands of the Adirondack Park, NY, 2007–2016. Dynamic rates were modeled as constant (as depicted) and with annual variability (not shown).

Model	Predicted dynamics dependent on
ψ (.), γ(.), ε(.)	Constant rates of colonization and extinction.
ψ (.), γ(Winter Temp), ε(.)	Colonization driven by mean winter temperature.
ψ (.), γ(Breeding Temp), ε(.)	Colonization driven by mean breeding season temperature.
ψ (.), γ(Var Winter Temp), ε(.)	Colonization driven by winter temperature variability.
ψ (.), γ(Var Breeding Temp), ε(.)	Colonization driven by breeding season temperature variability.
ψ (.), γ(Winter Ppt), ε(.)	Colonization driven by mean winter precipitation.
ψ (.), γ(Breeding Ppt), ε(.)	Colonization driven by mean breeding season precipitation.
ψ (.), γ(Var Winter Ppt), ε(.)	Colonization driven by winter precipitation variability.
ψ (.), γ(Var Breeding Ppt), ε(.)	Colonization driven by breeding season precipitation variability.
ψ (.), γ(Temp Hottest Month), ε(.)	Colonization driven by mean temperature of hottest month.
ψ (.), γ(Temp Coldest Month), ε(.)	Colonization driven by mean temperature of coldest month.
ψ (.), γ(Ppt Wettest Month), ε(.)	Colonization driven by mean precipitation of wettest month.
ψ (.), γ(Ppt Driest Month), ε(.)	Colonization driven by mean precipitation of driest month.
ψ (.), γ(.), ε(Winter Temp)	Extinction driven by mean winter temperature.
ψ (.), γ(.), ε(Breeding Temp)	Extinction driven by mean breeding season temperature.
ψ (.), γ(.), ε(Var Winter Temp)	Extinction driven by winter temperature variability.
ψ (.), γ(.), ε(Var Breeding Temp)	Extinction driven by breeding season temperature variability.
ψ (.), γ(.), ε(Winter Ppt)	Extinction driven by mean winter precipitation.
ψ (.), γ(.), ε(Breeding Ppt)	Extinction driven by mean breeding season precipitation.
ψ (.), γ(.), ε(Var Winter Ppt)	Extinction driven by winter precipitation variability.
ψ (.), γ(.), ε(Var Breeding Ppt)	Extinction driven by breeding season precipitation variability.
ψ (.), γ(.), ε(Temp Hottest Month)	Extinction driven by mean temperature of hottest month.
ψ (.), γ(.), ε(Temp Coldest Month)	Extinction driven by mean temperature of coldest month.
ψ (.), γ(.), ε(Ppt Wettest Month)	Extinction driven by mean precipitation of wettest month.
ψ (.), γ(.), ε(Ppt Driest Month)	Extinction driven by mean precipitation of driest month.

### Combined climate and non-climate drivers

We ran a full set of models for each species combining model sets from Parts 1 and 2 of our analysis to examine non-climate drivers (wetland size, connectivity, latitude, elevation, and human footprint) in combination with temperature and precipitation characteristics in order to assess the relative contribution of both to long-term boreal bird dynamics in the Adirondack Park. Because results from Part 2 of this analysis (Results) indicated that dynamic rates were influenced by average rather than yearly temperature and precipitation characteristics, in this final model set we included the effect of average rather than annual temperature and precipitation conditions on colonization and extinction across the 10-year study period.

## Results

### Detection probabilities

Both the prior 5-year [11] and the present 10-year analysis began with an analysis of detection probability which then informed all subsequent models for each of our boreal bird species. As per the previous analysis, no single variable best explained detection across all species. Among our 8 target species, covariates of observer and sky code (relative cloud cover) were represented most often in top models for detection but there was also support for the influence of temperature, time of day, date, and wind conditions. In general, detection probability was increased with use of expert observers (as per [11]); included in top models for all species except the flycatchers), and declined with increases in Julian date (yellow-bellied flycatcher), time of day (olive-sided flycatcher), temperature (Lincoln’s sparrow and palm warbler), and cloud cover (boreal, chickadee, Canada jay, rusty blackbird).

### Non-climate drivers

In general, results of the 10-year analysis agreed with and confirmed prior findings from the analysis of the 5-year dataset. Wetland area, connectivity, and human footprint remained the most consistent drivers across species in terms of the direction of influence, with the majority of species more likely to persist in large, well connected sites with low human footprint (Table 3). In contrast, elevation and latitude were inconsistent across species with respect to the direction of influence, but were important in terms of cumulative model weights (Table 3). Latitude was the most important factor in terms of cumulative model weight with respect to colonization probability and second only to human footprint with respect to extinction probability. The direction of this relationship, however, was not uniform: five species demonstrated a positive colonization response to increasing latitude, while three had a negative relationship. Elevation was important to colonization probability but much less so to extinction probability. Elevation had a nearly uniformly positive relationship with extinction, with 7/8 species displaying increasing extinction rates at higher elevations. These results are not entirely unexpected as higher latitude sites in our system correspond to lower elevations and other favorable conditions. The “boreal core” sites in our system are also located at high latitude. These sites include several of the largest and most well-connected wetland complexes which also tend to have low levels of human footprint. Latitude was among top models for 5 species, while wetland area, connectivity, elevation, and human footprint were among top models for 2–3 species each (Table 3).

**Table 3:** Summary of model weights (AIC weight for model containing each covariate) and selection results from analysis of underlying dynamics for 8 bird species monitored in boreal wetlands in the Adirondack Park, NY, 2007–2016. Cumulative weight indicates sum across species of all models containing the covariate (factor weight). Bold denotes that the covariate was included in top models (ΔAIC ≤ 2.0) for the species; shading indicates a positive influence of covariate on dynamic rates of colonization and/or extinction.

Covariate	BBWO	BOCH	CAJA	LISP	OSFL	PAWA	RUBL	YBFL	Cum. Wt.
	Colonization Factors								
Wetland Area	**0.13**	0.07	0.06	0.07	0.05	0.02	**0.16**	0.02	0.57
Connectivity	0.02	0.02	**0.57**	0.06	**0.08**	0.04	0.04	0.03	0.87
Latitude	**0.25**	**0.22**	0.01	**0.24**	0.04	0.10	**0.39**	0.03	1.28
Elevation	0.08	0.04	0.04	0.05	0.05	**0.49**	0.12	0.02	0.89
Human Footprint	0.01	0.04	0.01	0.07	0.04	0.03	0.04	0.04	0.27
	Extinction Factors								
Wetland Area	**0.11**	0.02	0.04	0.06	**0.14**	0.05	0.03	0.02	0.46
Connectivity	**0.18**	0.04	0.13	0.05	0.04	0.05	0.05	0.03	0.58
Latitude	**0.20**	**0.44**	0.01	0.07	**0.22**	0.06	0.04	0.03	1.05
Elevation	0.02	0.02	0.04	0.07	**0.17**	0.07	0.03	0.02	0.43
Human Footprint	0.01	0.04	0.08	**0.11**	0.05	0.03	0.03	**0.04**	1.11

We calculated occupancy trends over the 10-year study period in order to determine whether trends identified in the prior 5-year analysis had continued. We had previously identified patterns of declining occupancy for black-backed woodpecker, boreal chickadee, and olive-sided flycatcher, as well as a decline, though small, for Canada jay. Lincoln’s sparrow and rusty blackbird showed little change in occupancy rates over the prior 5-year analysis period, while palm warbler was the sole species with a demonstrable increase in occupancy [11]. We did not observe any reversals among these patterns. As before, black-backed woodpecker, boreal chickadee, and olive-sided flycatcher showed declines, with the decline for boreal chickadee appearing to be much steeper than the other species. A pattern of decline was newly observed for Lincoln’s sparrow and yellow-bellied flycatcher. Rusty blackbird also showed a small decline and already exists at very low levels of occupancy in this landscape. Updated trends showed that Canada jay appeared to be stable and, again, palm warbler remained the only species with an appreciably increasing occupancy pattern (Table 4).

**Table 4:** Model-averaged parameter estimates of occupancy (ψ), colonization (γ), extinction (ε), and growth rate (λ, calculated as the geometric mean of the λ’s for 2008–2016) for 8 boreal bird species monitored in the Adirondack Park, NY 2007–2016.

Parameter	BBWO	BOCH	CAJA	LISP	OSFL	PAWA	RUBL	YBFL
Ψ_2007_	0.80±0.11	0.47±0.16	0.67±0.12	0.65±0.07	0.66±0.12	0.43±0.07	0.22±0.12	0.87±0.06
Ψ_2016_	0.49±0.11	0.05±0.16	0.69±0.12	0.52±0.07	0.39±0.12	0.58±0.07	0.18±0.12	0.60±0.06
γ	0.14±0.08	0.01±0.02	0.29±0.21	0.15±0.02	0.13±0.01	0.13±0.04	0.13±0.07	0.21±0.01
ε	0.15±0.04	0.28±0.16	0.13±0.02	0.14±0.01	0.22±0.04	0.09±0.01	0.59±0.22	0.15±0.10
λ	0.95	0.77	1.00	0.97	0.94	1.03	0.98	0.96

### Climate drivers

We found no support for models with year-to-year variation, suggesting that annual extinction and colonization rates may not track interannual variability in temperature and precipitation. Our subsequent results and discussion therefore refer only to the responses of species to average climate conditions over the course of the study, and thus relate to spatial patterns of temperature and precipitation and their influence on occupancy patterns. Factor weights (cumulative model weights across all species) revealed a number of patterns in the responses of birds to temperature and precipitation (Table 5). Colonization appeared to be driven more strongly by precipitation factors, whereas extinction was driven more predominantly by temperature characteristics. Additionally, breeding season characteristics appeared more influential on colonization processes, whereas winter appeared to be a more critical period with respect to extinction dynamics. Unsurprisingly, cumulative model weights for models incorporating winter temperature and precipitation variables were higher, on average, for resident birds than for migrant species. In general, average conditions and variability in average conditions were more important than extremes in temperature and precipitation, though extremes were somewhat important in terms of extinction dynamics. Among individual variables, colonization rates were most strongly influenced by breeding and winter season precipitation, while extinction rates were most strongly influenced by breeding season temperature, followed by winter precipitation (Table 5).

**Table 5:** Summary of model weights (AIC weight for model containing each covariate) and selection results from analysis of underlying dynamics for 8 bird species monitored in boreal wetlands in the Adirondack Park, NY, 2007–2016. Cumulative weight indicates sum across species of all models containing the covariate (factor weight). Bold denotes that the covariate was included in top models (ΔAIC ≤ 2.0) for the species; shading indicates a positive influence of covariate on dynamic rates of colonization and/or extinction.

Covariate	BBWO	BOCH	CAJA	LISP	OSFL	PAWA	RUBL	YBFL	Cum. Wt.
			Colonization Factors						
Winter Temp	0.00	0.01	0.01	0.03	0.02	0.00	0.01	0.00	0.08
Breeding Season Temp	0.00	0.04	0.01	0.02	0.02	0.00	0.03	0.00	0.12
Var Winter Temp	0.00	0.03	**0.05**	0.02	0.02	0.00	0.01	0.00	0.14
Var Breeding Temp	0.10	0.01	0.02	0.02	0.04	0.01	0.02	0.00	0.20
Temp Hottest Month	0.00	0.06	0.01	0.02	0.02	0.00	0.02	0.00	0.13
Temp Coldest Month	0.00	0.02	0.01	0.03	0.02	0.00	0.01	0.00	0.10
Winter Ppt	**0.52**	0.01	0.01	**0.08**	0.00	0.00	0.01	0.00	0.64
Breeding Season Ppt	0.00	0.01	0.03	0.01	0.03	**0.49**	**0.54**	0.00	1.12
Var Winter Ppt	0.08	0.02	0.04	0.03	0.02	0.06	0.02	0.00	0.27
Var Breeding Ppt	0.04	0.15	0.03	**0.16**	0.02	0.00	0.01	0.00	0.41
Ppt Wettest Month	0.00	0.01	0.03	0.03	0.03	0.00	0.01	0.00	0.10
Ppt Driest Month	0.00	0.01	0.01	0.01	0.03	**0.26**	0.12	0.00	0.45
			Extinction Factors						
Winter Temp	0.00	0.01	0.04	0.03	0.05	0.00	0.03	0.12	0.29
Breeding Season Temp	0.00	0.00	**0.12**	0.06	**0.07**	0.00	0.04	**0.52**	0.83
Var Winter Temp	0.00	0.08	**0.12**	0.01	0.03	0.00	0.02	0.00	0.27
Var Breeding Temp	0.01	0.01	0.01	0.02	0.03	0.00	0.02	0.00	0.09
Temp Hottest Month	0.00	0.00	**0.12**	**0.07**	**0.07**	0.01	0.02	**0.21**	0.50
Temp Coldest Month	0.00	0.00	0.04	0.06	**0.09**	0.00	0.03	0.12	0.35
Winter Ppt	**0.19**	**0.45**	0.02	0.02	0.04	0.01	0.00	0.00	0.73
Breeding Season Ppt	0.01	0.00	**0.09**	**0.08**	0.04	0.05	0.00	0.00	0.28
Var Winter Ppt	0.02	0.03	**0.06**	0.03	**0.17**	0.05	0.01	0.00	0.36
Var Breeding Ppt	0.01	0.03	0.01	0.01	0.02	0.00	0.01	0.00	0.09
Ppt Wettest Month	0.00	0.00	0.02	0.01	0.03	0.00	0.01	0.00	0.08
Ppt Driest Month	0.01	0.01	**0.05**	**0.09**	0.02	0.02	0.00	0.00	0.21

Responses to temperature and precipitation among individual species were highly variable. Five species were best described by 1 or 2 models (black-backed woodpecker, boreal chickadee, palm warbler, rusty blackbird, yellow-bellied flycatcher), but for 3 species (Canada jay, Lincoln’s sparrow, olive-sided flycatcher) a total of 4 to 7 models could be considered equally good representatives of dynamic rates (ΔAIC < 2.0). Among top models (Table 5), precipitation variables occurred 14 times, while temperature variables occurred in 10 top models. Breeding and winter precipitation, as well as mean temperature of the hottest month, occurred most often among top models (4 each), followed by breeding season temperature and mean precipitation of the driest month (3 each). Variability in winter temperature and precipitation, variability in breeding season precipitation, and mean temperature of the coldest month occurred in 1–2 top models. Only 3 of the 12 climate variables–mean winter temperature, variability in breeding season temperature, and mean precipitation in the wettest month–did not occur in top models for any species. Top models more often contained variables placed on extinction rates (17 models) than colonization rates (7 models).

Among variables occurring in top models, direction of influence on individual species’ colonization rates was highly variable (Table 5). Breeding season precipitation was included in top colonization models for palm warbler and rusty blackbird, but had opposite effects (negative influence on palm warbler colonization rates but positive influence for rusty blackbird). Winter season precipitation was important for black-backed woodpecker and Lincoln’s sparrow and positively affected colonization rates for both species. Among other factors included in top colonization models, variability in winter temperatures positively influenced colonization for Canada jay, while variability in breeding season precipitation negatively influenced colonization for Lincoln’s sparrow. Last, mean precipitation for the driest month was among top models for palm warbler and negatively influenced colonization rates.

Responses to temperature and precipitation were more consistent across species with respect to influence on extinction rates (Table 5). Mean breeding season temperatures were negatively associated with extinction probability (and therefore positively associated with persistence) for Canada jay, olive-sided flycatcher, and yellow-bellied flycatcher, the three species for which this variable was included in top models. Mean temperature of the hottest month was included in top models for the same 3 species as well as Lincoln’s sparrow and displayed a similar pattern, being a positive influence on persistence for all 4 species. Winter precipitation appeared in top models for 2 resident birds, the black-backed woodpecker and the boreal chickadee, having a positive and negative effect on their persistence, respectively. Across all species, in fact, winter precipitation positively influenced extinction rates (and therefore was negatively associated with persistence) for all but the black-backed woodpecker. Breeding season precipitation was included in top models for 2 species (Canada jay and Lincoln’s sparrow) and negatively influenced persistence in both cases. Two additional factors related to variability were included in top extinction models including variability in winter temperatures, which increased persistence of Canada jay, and variability of winter precipitation, which decreased persistence of Canada jay and olive-sided flycatcher. Last, two variables related to extreme conditions were included in top models including mean temperature of the coldest month, which increased persistence of olive-sided flycatcher, and mean precipitation of the driest month, which decreased persistence of Canada jay and Lincoln’s sparrow. Though not included in top models for all species, two variables–mean winter temperature and mean temperature of the coldest month–showed a consistent influence across all 8 target species, increasing persistence in all cases (Table 5). Similarly, mean breeding season temperature increased persistence for all but one species (boreal chickadee), while both mean winter precipitation and variability in mean winter precipitation decreased persistence for all but one species (black-backed woodpecker). Consideration of mean temperature and precipitation alone (i.e., in the absence of variability and extremes) reveals that the net effect of these factors is variable across species but that, broadly, responses to temperature are generally positive or mixed, whereas responses to precipitation are largely negative (Table 6).

**Table 6:** Net effect of temperature and precipitation on species-specific colonization and extinction rates. Net effect on the species of both climate variables is described as: increased colonization and decreased extinction is considered *positive* (+); decreased colonization and increased extinction is considered *negative* (-); and conflicting relationships is considered *mixed* (o). To capture the overall net effect of directional climate change on species, we only include effects of changes in average temperature and precipitation (i.e., variability and extremes not considered).

Species	Breeding Season		Winter	
	Temperature	Precipitation	Temperature	Precipitation
BBWO	+	+	+	+
BOCH	-	o	+	-
CAJA	+	-	o	-
LISP	o	-	o	o
OSFL	+	-	o	o
PAWA	+	-	o	-
RUBL	+	o	+	-
YBFL	+	-	+	-

### Combined climate and non-climate drivers

The last part of our analysis combined models from Parts 1 and 2 to determine the degree to which climate influences extinction and colonization patterns relative to previously identified drivers. We found that for 6 of 8 target species, top models were climate driven (Table 7). Top models for one species included both climate and landscape context variables, and only one species (Canada jay) had top models containing only non-climate drivers.

**Table 7:** Summary of model selection results from analysis of underlying dynamics for 8 bird species monitored in boreal wetlands in the Adirondack Park, NY, 2007–2016. Covariates are explained in methods; only the results of top models are shown (ΔAIC ≤ 2.0).

Spp	Model	AIC	ΔAIC	AICwt	Likelihood	#Par	-2LogLike
BBWO	ψ (.), γ(Winter Ppt), ε(.), p(obs)	982.16	0	0.3746	1	6	970.16
	ψ (.), γ(.), ε(Winter Ppt), p(obs)	984.16	2	0.1378	0.3679	6	972.16
BOCH	ψ (.), γ(.), ε(Winter Ppt), p(obs,sky)	427.7	0	0.376	1	7	413.7
CAJA	ψ (.), γ(Connectivity), ε(.), p(obs,sky)	1054.89	0	0.3929	1	7	1040.89
LISP	ψ (.), γ(Var Breeding Ppt), ε(.), p(temp)	1803.46	0	0.1373	1	6	1791.46
	ψ (.), γ(.), ε(Ppt Driest Month), p(temp)	1804.59	1.13	0.078	0.5684	6	1792.59
	ψ (.), γ(.), ε(Breeding Ppt), p(temp)	1804.79	1.33	0.0706	0.5143	6	1792.79
	ψ (.), γ(.), ε(Winter Ppt), p(temp)	1804.82	1.36	0.0695	0.5066	6	1792.82
	ψ (.), γ(.), ε(Temp Hottest Month), p(temp)	1805.03	1.57	0.0626	0.4561	6	1793.03
OSFL	ψ (.), γ(.), ε(Var Winter Ppt), p(time)	852.6	0	0.1186	1	6	840.6
	ψ (.), γ(.), ε(Latitude), p(time)	853.56	0.96	0.0734	0.6188	6	841.56
	ψ (.), γ(.), ε(Temp Coldest Month), p(time)	853.8	1.2	0.0651	0.5488	6	841.8
	ψ (.), γ(.), ε(Elevation), p(time)	854.03	1.43	0.058	0.4892	6	842.03
	ψ (.), γ(.), ε(Breeding Temp), p(time)	854.29	1.69	0.051	0.4296	6	842.29
	ψ (.), γ(.), ε(Temp Hottest Month), p(time)	854.34	1.74	0.0497	0.419	6	842.34
	ψ (.), γ(.), ε(Wetland Area), p(time)	854.41	1.81	0.048	0.4045	6	842.41
PAWA	ψ (.), γ(Breeding Ppt), ε(.), p(obs,temp)	1624.85	0	0.4663	1	7	1610.85
	ψ (.), γ(Ppt Driest Month), ε(.), p(obs,temp)	1626.13	1.28	0.2459	0.5273	7	1612.13
RUBL	ψ (.), γ(Breeding Ppt), ε(.), p(obs,sky)	281.25	0	0.46	1	7	267.25
YBFL	ψ (.), γ(.), ε(Breeding Temp), p(obs,date)	2120.98	0	0.5135	1	7	2106.98
	ψ (.), γ(.), ε(Temp Hottest Month), p(obs,date)	2122.79	1.81	0.2077	0.4045	7	2108.79

**Fig 2:**
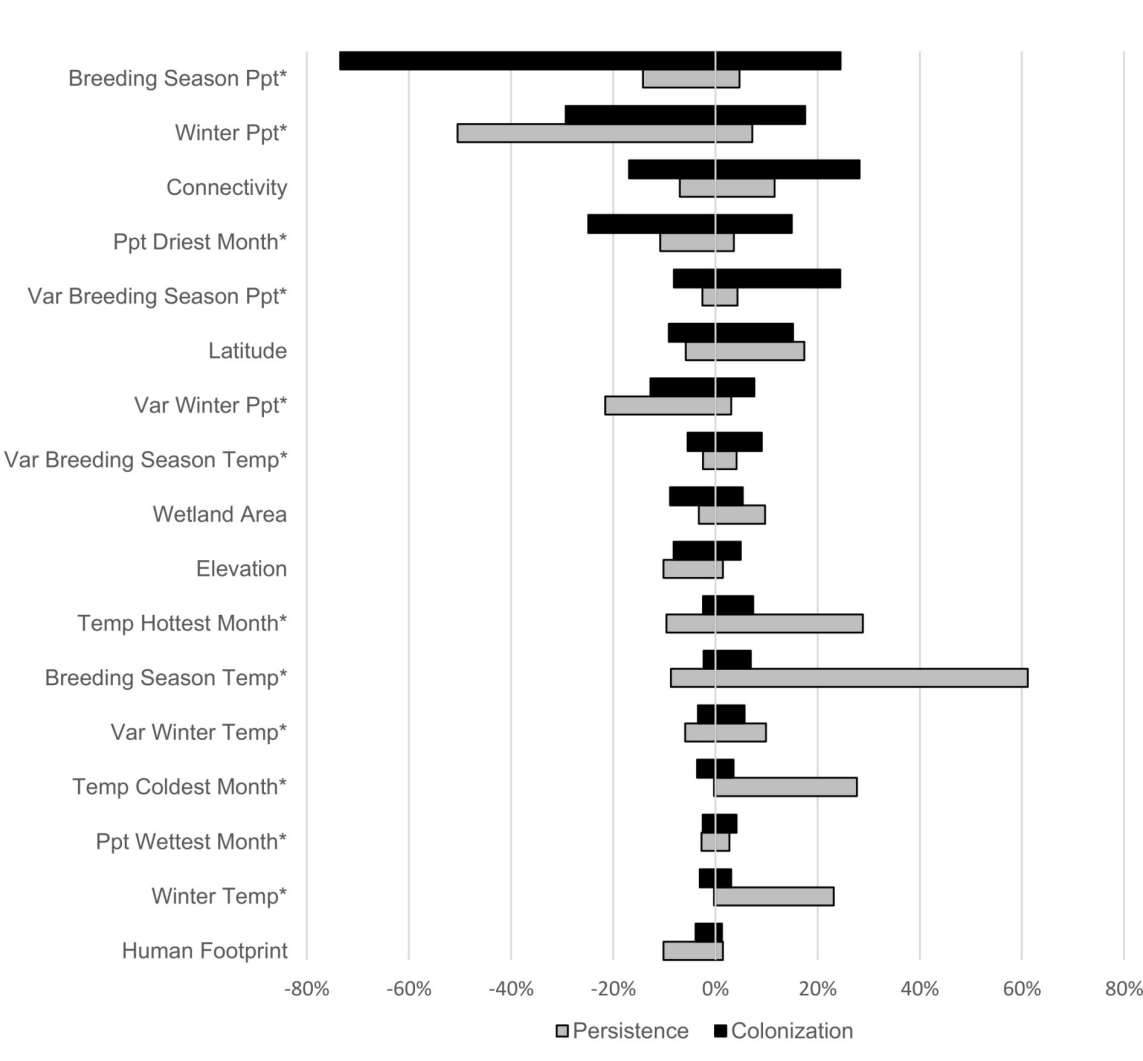
Factors influencing boreal bird colonization and persistence in the Adirondack Park, NY, 2007–2016. The size of each bar represents the cumulative model weight, summed across species, for factors affecting colonization and persistence (1—extinction) rates in Adirondack boreal wetlands. The proportion of the bar on either size of 0 indicates the proportion of species for which the influence of the factor was positive or negative. Breeding season precipitation, for example, was the most important influence on colonization rates, and negatively associated with colonization for most species. Climate variables are denoted by *.

Responses were variable across species, but with respect to colonization, breeding season precipitation had higher cumulative model weight across all species than did non climate factors and its influence was generally negative. Second most important to colonization rates was winter season precipitation, which was also generally a negative influence, followed by wetland connectivity. Similarly, with respect to extinction drivers, breeding season temperature and winter precipitation were more influential in controlling extinction rates than previously considered non-climate variables. Higher breeding season temperatures tended to decrease extinction probability while higher winter precipitation enhanced it. Consideration of all factors together demonstrates that boreal birds appear most likely to colonize sites that have generally lower levels of precipitation, and a high degree of connectivity, while they tend to persist in sites that are warmer in the breeding season and have low and less variable precipitation in the winter (Fig 2).

## Discussion

The Adirondack Park lies at the transition between the temperate and boreal biomes and provides habitat for a number of specialized and rare northern birds. As species at the southern edge of their range, they are expected to be vulnerable to climate change and potential indicators of the influence of climate change on wildlife in a high latitude system [25,26,27]. Our findings have confirmed patterns of declining occupancy identified previously [11] and revealed evidence of decline among additional species. Six of 8 target species exhibited patterns of decline in occupancy between 2007 and 2016, and only one species (palm warbler) demonstrates an increasing trend. These findings are similar to those from other studies and regions [28,13,29,30] and suggest that both flycatcher species, as well as the already sparse rusty blackbird may be of particular concern in the Adirondack landscape.

We previously highlighted the importance of several non-climate factors in driving occupancy patterns, including latitude and elevation. Responses to latitude and elevation may reflect possible northward or upslope movements influenced by climate change as temperatures warm. Our prior analysis identified mixed responses, with most species more likely to persist at low elevation and high latitude, which only partially supports the expectation of northward and upward movement in response to climate change. However, our study focused solely on low elevation boreal community types and, among them, increasing latitude is associated with lower elevation. The current analysis demonstrates the degree to which temperature and precipitation may offer a better means of exploring the influence of climate than latitude and elevation, as temperature and precipitation patterns appear to be more closely related to extinction and colonization dynamics for most species.

Though responses were highly species specific, we found that, in general, extinction and colonization processes were both important in controlling long-term dynamic occupancy patterns for boreal birds and that extinction was related most closely to temperature patterns, whereas colonization appears to be more closely related to precipitation. Across all factors and all species, persistence appears most likely at sites characterized by warmer average temperatures and low precipitation (especially in winter), combined with large wetland size, high connectedness, and low human footprint. These characteristics are found most often among our study sites in the northwest “boreal core” of the Adirondacks (Fig 1). The largest and most well-connected open peatlands are at high latitudes and low elevations in the northwest quadrant of the park. These locations experience the lowest levels of precipitation in the park; precipitation is generally highest in a band running approximately northeast to southwest and associated with elevation. These drier sites in the boreal core, which also tend to be large and characterized by relatively low human footprint, may represent refugia for boreal species. The large open peatlands are also characterized by higher mean temperatures than sites with larger forested components.

### Relevance to projected changes in temperature and precipitation

Ongoing and projected climate change patterns in the northeastern US indicate a future that is decidedly warmer, especially in the winter months [31,32]. Increasing temperatures may benefit birds in the short term through increases in insect food supplies [33,34,35] and may also increase nest success for some species [36,37,38]. We found that temperature demonstrated a highly consistent influence across boreal bird species and was positively associated with persistence for all species with respect to winter temperatures and all but one species with respect to the breeding season. The high relative importance of temperature for persistence, however, is due almost entirely to its influence on yellow-bellied flycatcher. Yellow-bellied flycatcher, a ground nesting species, may benefit in particular from increased nestling survivorship or increased abundance of flying insects that may be associated with warmer mean temperatures.

Predictions about future precipitation in the northeast are less certain but generally indicate an increase in winter precipitation, with a greater proportion occurring as rain rather than snow, and increased variability in precipitation patterns overall [31,32]. We found that precipitation, particularly in the breeding season, was important to colonization dynamics of boreal birds, whereas winter precipitation influenced both colonization and extinction probabilities. Increased winter precipitation may have negative effects on persistence of some species, especially those which are present year round. For two resident species–boreal chickadee and Canada jay–the influence of winter precipitation appeared to be primarily negative, while black-backed woodpecker responded positively to winter precipitation. Future changes in summer precipitation are more uncertain than those in winter, and we found mixed effects of breeding season precipitation on colonization. Higher breeding season precipitation was negatively associated with persistence for most species, and variability of precipitation during the breeding season had mixed effects. Variability in winter precipitation increased extinction probability for all species except black-backed woodpecker. Precipitation is, in general, much more variable than temperature in our study locations. The mean highest and lowest temperature months occur reliably at the same time of year annually, but mean driest and wettest months are extremely variable. Such variability may buffer migrant species from impacts of extreme precipitation events, given that 5 of 8 of the target species are absent from our study locations for a portion of the year.

### Implications

There is uncertainty as to the degree to which wildlife populations respond to average conditions with respect to climate, or whether variability and/or extremes of climate are more critical [39,40]. Though extremes and variability in temperature and precipitation patterns did affect some species strongly, we generally found more support for the importance of average conditions over time. We also found more support for constant rates of colonization and extinction which–though influenced by variable climate characteristics among sites–did not track annual levels of temperature and precipitation. It is possible that longer-term data collection will reveal such patterns eventually, as may other population parameters such as abundance or survival. These species may operate as metapopulations, given their occupancy of a naturally patchy habitat type in our region [11], and occupancy of spatially disjunct wetland habitats may reduce the level of agreement between abundance and occupancy patterns [41].

Though observed patterns of decline among boreal birds at the range margin are consistent with expectations based on climate change [42], direct relationships with temperature and precipitation do not follow predicted patterns for all species. This discrepancy may be partially accounted for by the geography of the Adirondack lowland boreal and the location of the largest open peatland complexes in the northwest core. This region of the park experiences relatively low levels of precipitation, and open peatland habitats are characterized by higher average temperatures than sites with higher proportions of forest and fen vegetation. These large peatlands are, interestingly, both warmer and colder than other boreal habitats with respect to daily maxima and minima. Ground level temperature data from several Adirondack boreal wetlands reveal both minimums and maximums of temperature that are a few degrees lower and higher, respectively, than predicted temperatures from PRISM data (Supporting information S1 File, S2 File). The lack of forest cover in these sites may allow for daily and seasonal temperature fluctuations to be more pronounced; essentially the extremes may be more extreme in these locations [43]. The lack of forest cover may also be associated with lower densities of red squirrel predators and of potential competitors including more cosmopolitan forest birds like blue jays. Both predators and potential competitors may be less adapted to the more extreme characteristics of these low nutrient, low productivity environments.

The future of large open peatlands in a warmer and wetter world is uncertain [4], and observed patterns of boreal bird occupancy may indicate a potential lag in the response of species to changing environmental conditions. A combination of warming temperatures and increased frequency of drought conditions during the summer months [44] has the potential to result in rapid tree encroachment into currently open boreal peatland systems [45,46,47]. The associated changes in habitat structure and temperature and hydrologic regimes may make them less favorable habitats for boreal avian species in the future. Is it possible that current positive associations of boreal birds with warmer site conditions reflect short term benefits such as increased insect prey abundance or nest survivorship; benefits that are likely to change over the long term as altered temperature and precipitation regimes result in fundamental changes to boreal plant communities.

Our findings suggest that, currently, the large and well-connected peatland complexes in the northwest region of the Adirondacks may serve as source habitats for metapopulations of boreal birds. Metapopulations, however, rely on the exchange of individuals between the sources and the sinks [48]. Most wetlands are small and isolated, and therefore most of the population occurs in sinks rather than sources [49,50]. Less favorable conditions in the sink habitats (more isolated, smaller, and more highly impacted) combined with increased and more variable precipitation in the future may further contribute to the contraction of these birds within the park toward the boreal core and away from boreal habitats in the northeast and southwest of the park, a pattern previously exhibited by spruce grouse, which was also once common in throughout boreal habitats in the Adirondacks [51]. Such contraction would be manifested in northward movement from southern boreal habitats, but also in westward and some southward movement from more marginal boreal habitats on the periphery of the boreal core. It would also serve to further isolate these species from their conspecifics in the Canadian boreal. Careful management of boreal wetlands in the Adirondacks, both large and small, will be essential to buffering the harmful effects of climate change on these characteristically northern habitats and species.

## Supporting information

S1 File Ground level temperature records from large open peatlands. (XLSX)

S2 File Explanatory information for ground level temperature records and comparison to PRISM data. (PDF)
