# Supplementary material for: Relative contribution of climate and non-climate drivers in determining dynamic rates of boreal birds at the edge of their range
Source: PLoS One. 2019 Oct 24;14(10):e0224308. doi: 10.1371/journal.pone.0224308 (PMC6812788; doi:10.1371/journal.pone.0224308)
Supplement: S2 File — (PDF) [file pone.0224308.s002.pdf]

Supplementary Data for Climate Change and Boreal Birds and their Range Margins

January 8, 2018

Stephen F. Langdon, M.S.

Shingle Shanty Preserve and Research Station

Methods

Locations

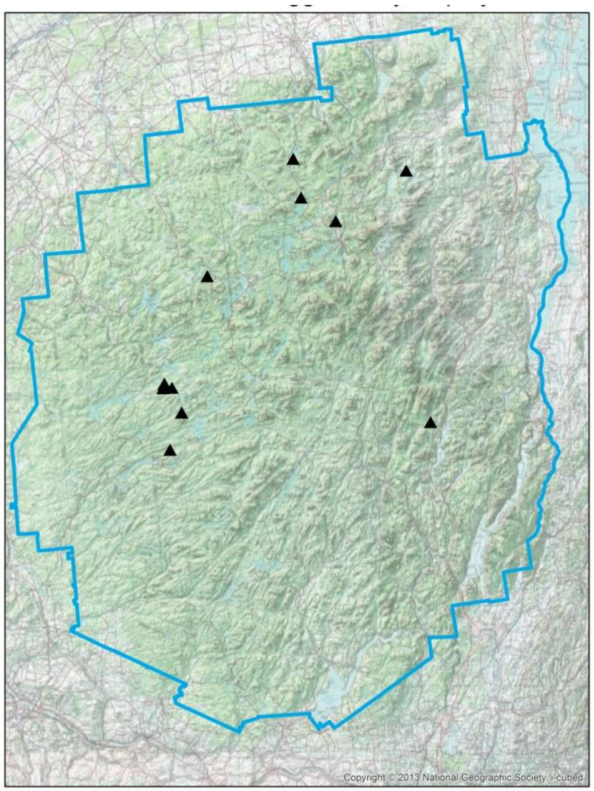

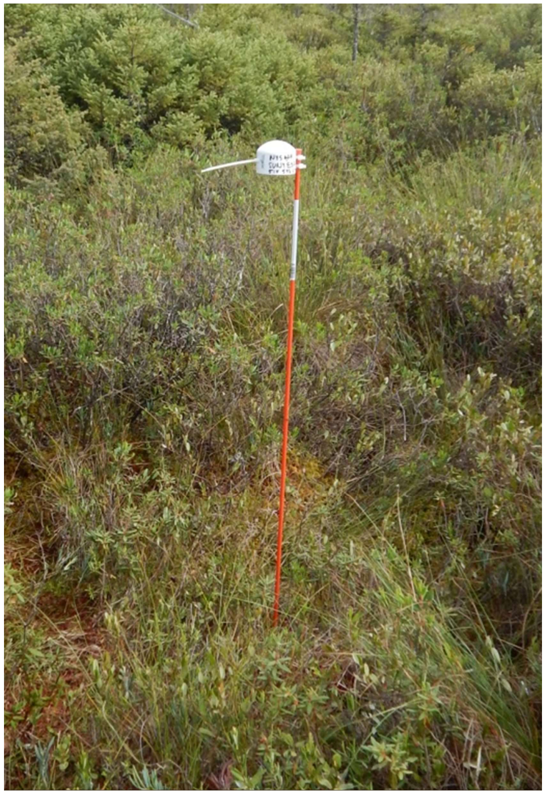

Methods

- Temperature data logger arrays were deployed at 13 sites at 1 m above the ground in unforested peatland communities.
- Temperature was measured every 2 hours.
- Duration Fall 2015 – Fall 2016 (since 2012 at Shingle Shanty)

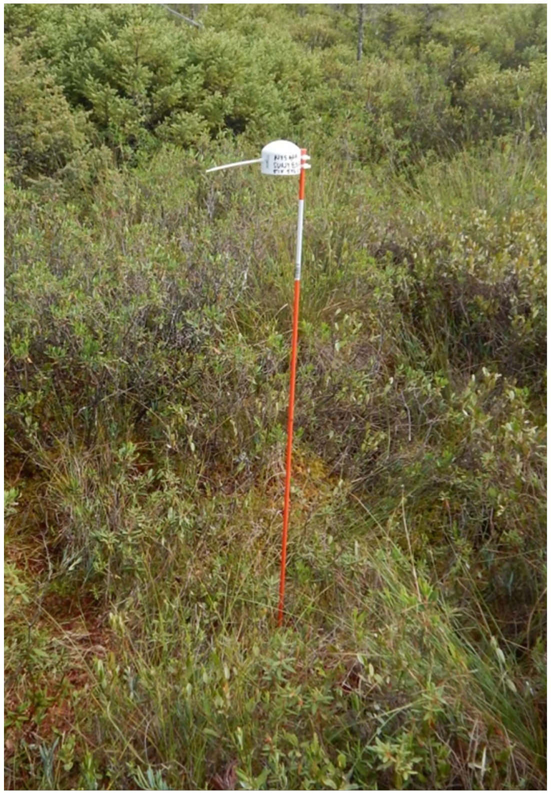

Methods

- Spatial Temperature Models PRISM
  - 800 m gridded monthly temp data
  - 4 km gridded daily temp data
- Hobo Pendant data loggers

http://www.prism.oregonstate.edu/

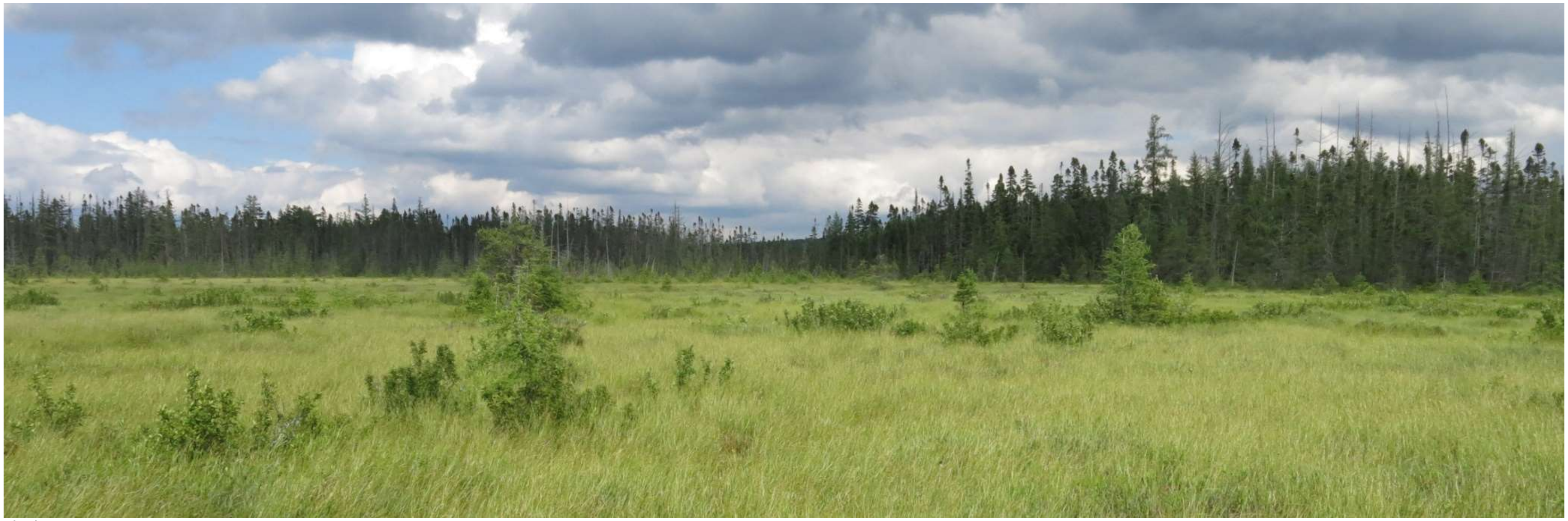

Results: What is the difference in modeled and measured monthly temperature values?

Tmin is 2° C colder

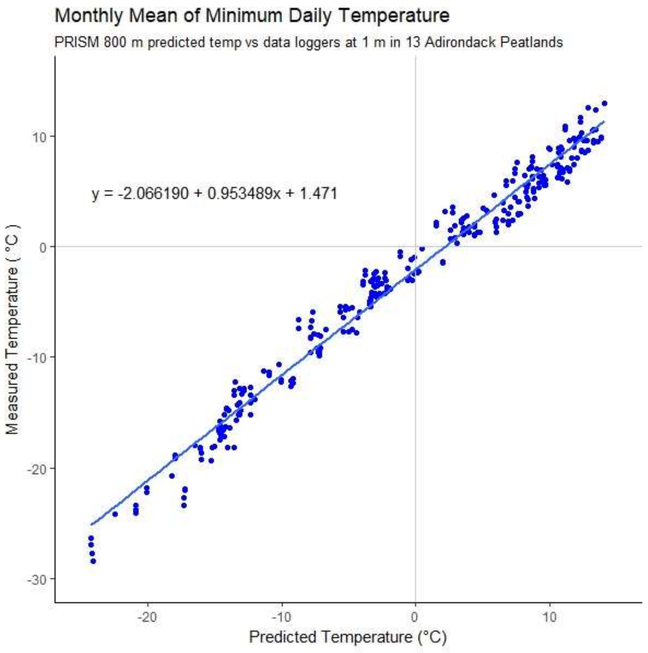

Tmean is 1° C warmer

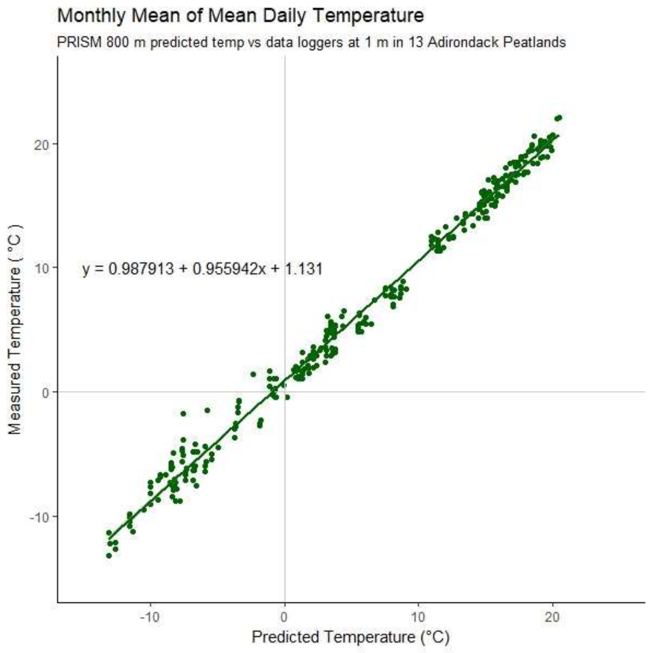

Tmax is 3.5° C warmer

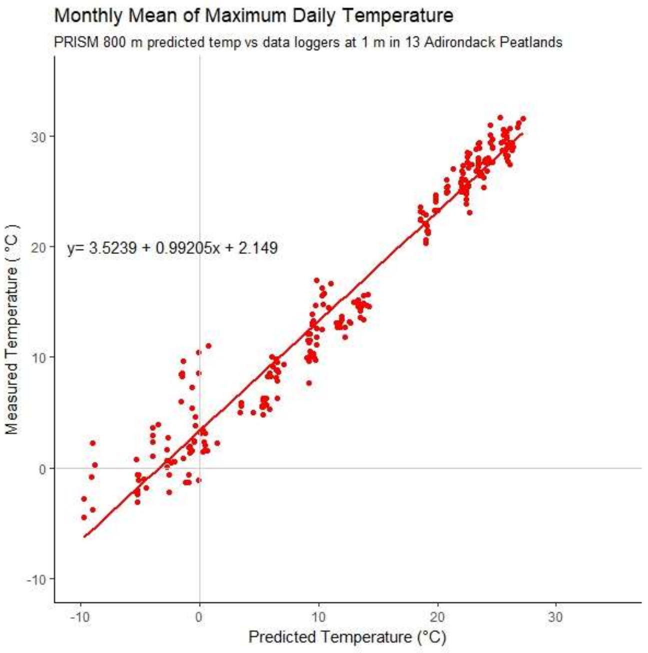

Results: Are some peatlands colder/warmer than others?

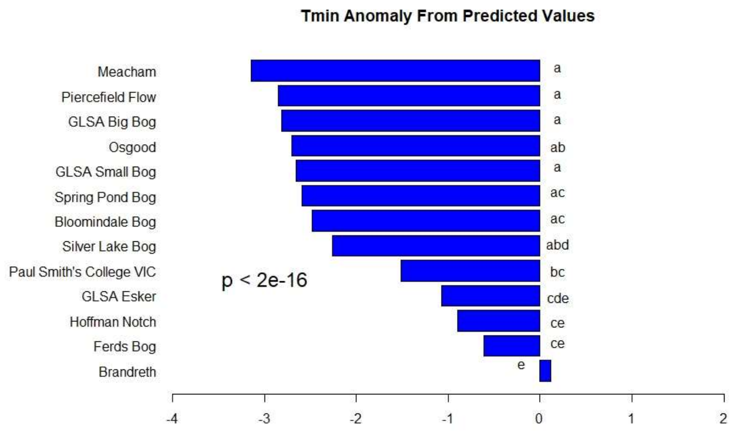

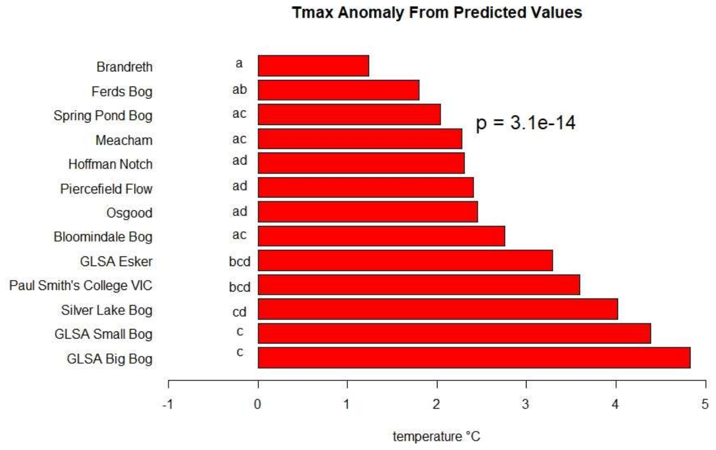

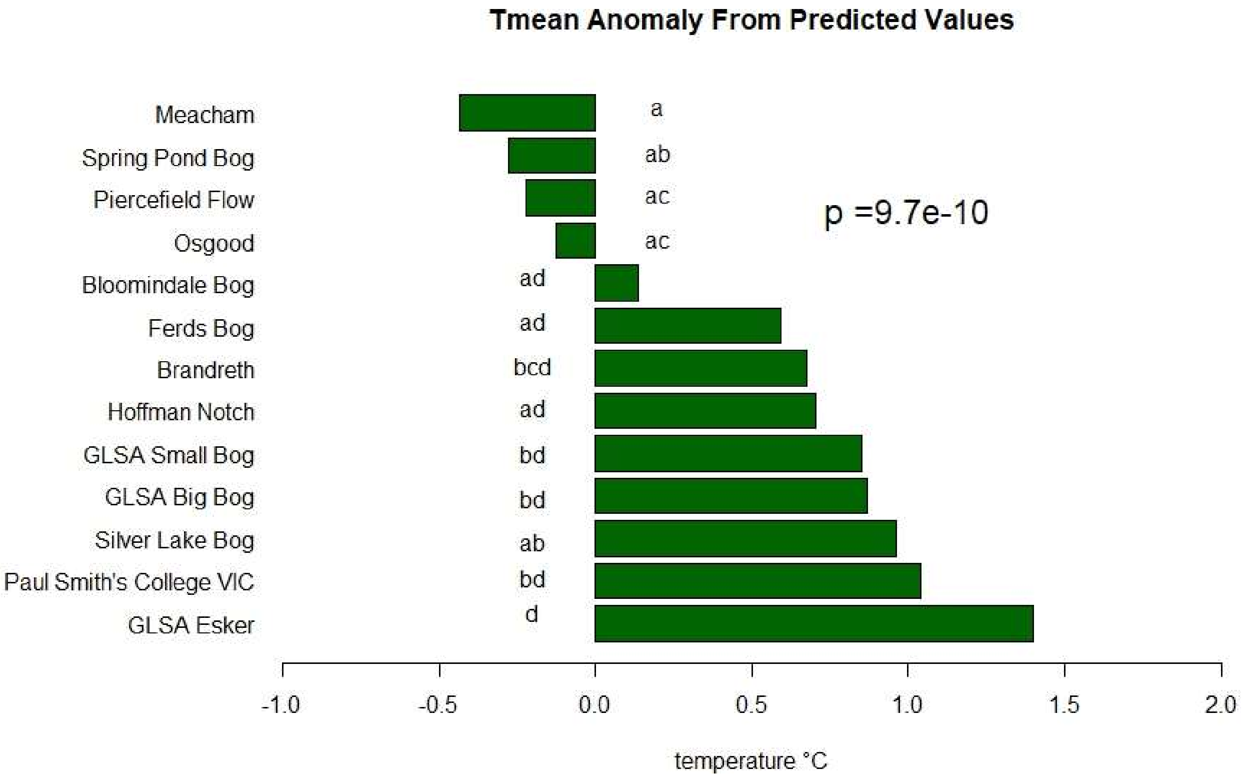

Results: How well do *daily* temperature models predict length of growing season in Peatlands?

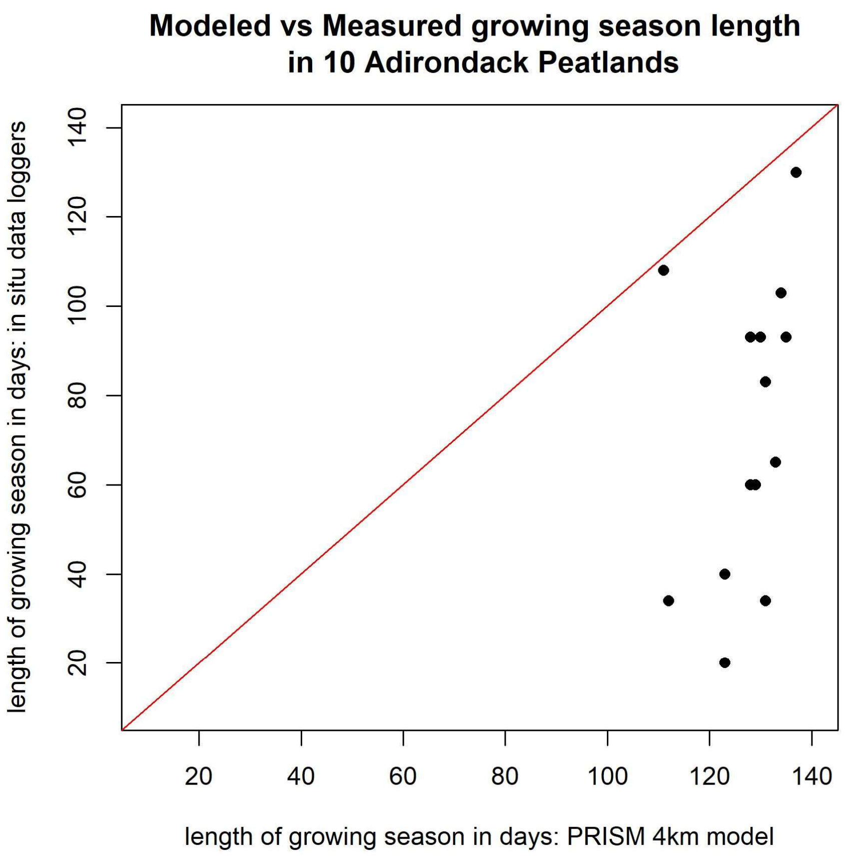

Acknowledgements

SSPRS

NECSC
Northeast Climate Science Center

EPA United States Environmental Protection Agency
Assistant Agreement No. #CD96295000
